# Automated Diagnosis of Knee Osteoarthritis Using ResNet101 on a DEEP:PHI: Leveraging a No-Code AI Platform for Efficient and Accurate Medical Image Analysis

**DOI:** 10.3390/diagnostics14212451

**Published:** 2024-11-01

**Authors:** Kyu-Hong Lee, Ro-Woon Lee, Jae-Sung Yun, Myung-Sub Kim, Hyun-Seok Choi

**Affiliations:** 1 Department of Radiology, Inha University College of Medicine, 27 Inhang-ro, Jung-gu, Incheon 22332, Republic of Korea; mdcappuccino@daum.net; 2 Department of Radiology, Ajou University School of Medicine, Suwon 16499, Republic of Korea; 3 Department of Radiology, Kangbuk Samsung Hospital, Sungkyunkwan University School of Medicine, Seoul 03181, Republic of Korea; 4 Deepnoid Inc., Seoul 08376, Republic of Korea

**Keywords:** no-coding, osteoarthritis, artificial intelligence

## Abstract

**Background:** Knee osteoarthritis (OA) is a prevalent degenerative joint disease significantly impacting global health. Early and accurate diagnosis is crucial for effective management, but traditional methods often rely on subjective assessments. This study evaluates the efficacy of a deep learning model implemented through a no-code AI platform for diagnosing and grading knee OA from plain radiographs. **Methods:** We utilized the Osteoarthritis Initiative (OAI) dataset, comprising knee X-ray data from 1526 patients. The data were split into training (47.0%), validation (26.5%), and test (26.5%) sets. We employed a ResNet101 model on the DEEP:PHI no-code AI platform for image analysis. The model was trained to classify knee OA into five grades (0–4) based on the Kellgren–Lawrence scale. **Results:** Our AI model demonstrated high accuracy in distinguishing between different OA grades, with particular strength in early-stage detection. The model achieved optimal performance at 20 epochs, suggesting efficient learning dynamics. Grad-CAM visualizations were used to enhance the interpretability of the model’s decision-making process. **Conclusions:** This study demonstrates the potential of AI, implemented through a no-code platform, to accurately diagnose and grade knee OA from radiographs. The use of a no-code AI platform such as DEEP:PHI represents a step towards democratizing AI in healthcare, enabling the rapid development and deployment of sophisticated medical AI applications without extensive coding expertise. This approach could significantly enhance the early detection and management of knee OA, potentially improving patient outcomes and streamlining clinical workflows.

## 1. Introduction

Osteoarthritis (OA) of the knee is a prevalent degenerative joint disease that significantly impacts the quality of life for millions of individuals worldwide [1]. As the global population ages, the incidence of knee OA is expected to rise, placing an increasing burden on healthcare systems. An early and accurate diagnosis of knee OA is crucial for effective management and improved patient outcomes. Traditional diagnostic methods for knee osteoarthritis (OA) using X-rays have several limitations. While X-rays are accessible and cost-effective, they mainly show bone structures and struggle to detect early OA changes like cartilage damage and soft tissue issues. They cannot directly visualize soft tissues like the synovium, making them less effective at identifying early cartilage degeneration and inflammation. Relying on joint space narrowing and osteophyte formation as signs of OA can be misleading, as these may not accurately reflect the severity of cartilage loss or symptoms. Additionally, X-rays are not sensitive to short-term changes, limiting their use in tracking disease progression or assessing treatment effectiveness. These limitations underscore the need for more advanced imaging techniques.

In recent years, artificial intelligence (AI) has emerged as a promising tool in medical diagnostics, offering the potential to enhance accuracy, efficiency, and consistency in disease identification and assessment. The application of AI in radiology and orthopedics has shown particularly encouraging results, with machine learning algorithms demonstrating the ability to analyze medical images with high precision [2]. However, the implementation of AI solutions in clinical practice has been hindered by the complexity of developing and deploying these systems, often requiring significant coding expertise and computational resources.

Recent advances in no-code AI platforms have significantly expanded their capabilities, making AI technology more accessible and versatile across various industries. These platforms now offer user-friendly drag-and-drop interfaces, allowing individuals without coding expertise to efficiently develop AI applications. For example, platforms like BuildFire AI and Builder.AI provide tools for creating mobile apps and AI-driven solutions with ease, leveraging pre-built templates and extensive libraries to streamline the development process. Additionally, no-code AI tools have integrated advanced functionalities such as natural language processing and computer vision, enabling users to build sophisticated applications without needing a team of data scientists [3]. The market for no-code AI platforms is rapidly growing, driven by the demand for rapid prototyping, cost efficiency, and enhanced collaboration among non-technical users. These advancements are democratizing AI development, empowering businesses to innovate quickly and effectively while reducing reliance on specialized technical skills [4].

Among them, DEEP:PHI represents a novel approach to addressing this challenge by offering a no-coding AI solution that aims to democratize access to advanced diagnostic tools. This platform allows healthcare professionals to harness the power of AI for medical image analysis without the need for extensive programming knowledge or specialized IT infrastructure (https://www.deepnoid.com/ai-platform?lang=en, accessed on 3 May 2024). The potential of DEEP:PHI to facilitate the diagnosis of knee OA presents an exciting opportunity to improve patient care and streamline clinical workflows.

This study aims to evaluate the efficacy and accuracy of DEEP:PHI in diagnosing knee osteoarthritis through the analysis of medical imaging data. By comparing the performance of this AI-driven approach with traditional diagnostic methods, we seek to assess its potential as a reliable and accessible tool for healthcare providers. Additionally, this research will explore the implications of integrating no-coding AI solutions like DEEP:PHI into clinical practice, considering factors such as ease of use, scalability, and impact on diagnostic accuracy. Moreover, this study includes considerations of how such technologies may augment clinical decision making, improve patient outcomes, and potentially reshape the landscape of medical diagnostics in the era of digital health.

## 2. Materials and Methods

### 2.1. Dataset

The data used to prepare this article were obtained from the Osteoarthritis Initiative (OAI) database, which is available for public access at https://data.mendeley.com/datasets/56rmx5bjcr/1, accessed on 3 May 2024 [5]. This dataset contains knee X-ray data of 895 patients, for knee joint osteoarthritis detection and knee Kellgren–Lawrence (KL) grading scale. The grade descriptions are as follows:

Grade 0: healthy knee image.

Grade 1 (doubtful): doubtful joint narrowing with possible osteophytic lipping.

Grade 2 (minimal): definite presence of osteophytes and possible joint space narrowing.

Grade 3 (moderate): multiple osteophytes, definite joint space narrowing, with mild sclerosis.

Grade 4 (severe): large osteophytes, significant joint narrowing, and severe sclerosis.

The OAI serves as the foundation for this longitudinal cohort investigation into knee osteoarthritis [4]. Comprehensive details regarding specific eligibility criteria and ethical considerations can be found within the Osteoarthritis Initiative Study Protocol [6]. It is important to note that the data utilized in crafting this article are publicly accessible through the OAI database and are not subject to proprietary restrictions.

The OAI provided ethical clearance for the collection of subject information, and each participant involved in this study granted their informed consent. It should be emphasized that this article does not encompass any research involving human subjects conducted by the authors themselves. Furthermore, all contributing authors have committed to an OAI data use agreement.

To ensure compliance with ethical standards, this research has received approval from the Inha University Hospital institutional review board committee on research ethics. The approval details are as follows: IRB No: 2024-04-002, with the date of approval being 24 April 2024.

### 2.2. Dataset Distribution

Our study encompassed a cohort of 1526 patients, stratified into 3 subsets: a training set (*n* = 717, 47.0%), a validation set (*n* = 405, 26.5%), and a test set (*n* = 404, 26.5%). The patient population was further categorized according to the Kellgren–Lawrence grading scale, resulting in the following distribution: 604 patients classified as grade 0, 275 as grade 1, 403 as grade 2, 200 as grade 3, and 44 as grade 4 (Table 1). These data were subsequently utilized to train our deep learning algorithms.

### 2.3. Image Preprocessing and AI-Learning Methodology

#### 2.3.1. Image Preprocessing

Our preprocessing pipeline for the plain radiographs of knee joints involved several key steps.

1. Resizing: Images were standardized to 256 × 256 pixels.

This standardization reduces computational demands and speeds up both training and inference by providing a manageable and uniform input size

2. Grayscale Conversion: Color information was removed to focus on structural details.

This reduction in data complexity helps highlight important features, particularly in medical imaging, where structural variations are more critical than color differences.

3. Z-score Standardization: This step ensured consistency in the dynamic range, potentially enhancing computational efficiency.

It enhances computational efficiency by stabilizing the learning process, making it easier for the model to converge during training

4. Contrast Limited Adaptive Histogram Equalization (CLAHE): This technique was applied to improve image contrast and achieve uniform histogram distribution, thereby accentuating image features crucial for analysis.

This technique highlights important features and improves local contrast, which is crucial for detecting subtle differences in medical images that may indicate disease.

#### 2.3.2. AI Model Architecture and Model Training Protocol

We employed the ResNet101 architecture for our deep learning model. ResNet101, a variant of the Residual Network family, is a convolutional neural network pretrained on the ImageNet database. It comprises 101 layers and was developed to investigate the impact of network depth on deep learning performance.

**Figure 1:**
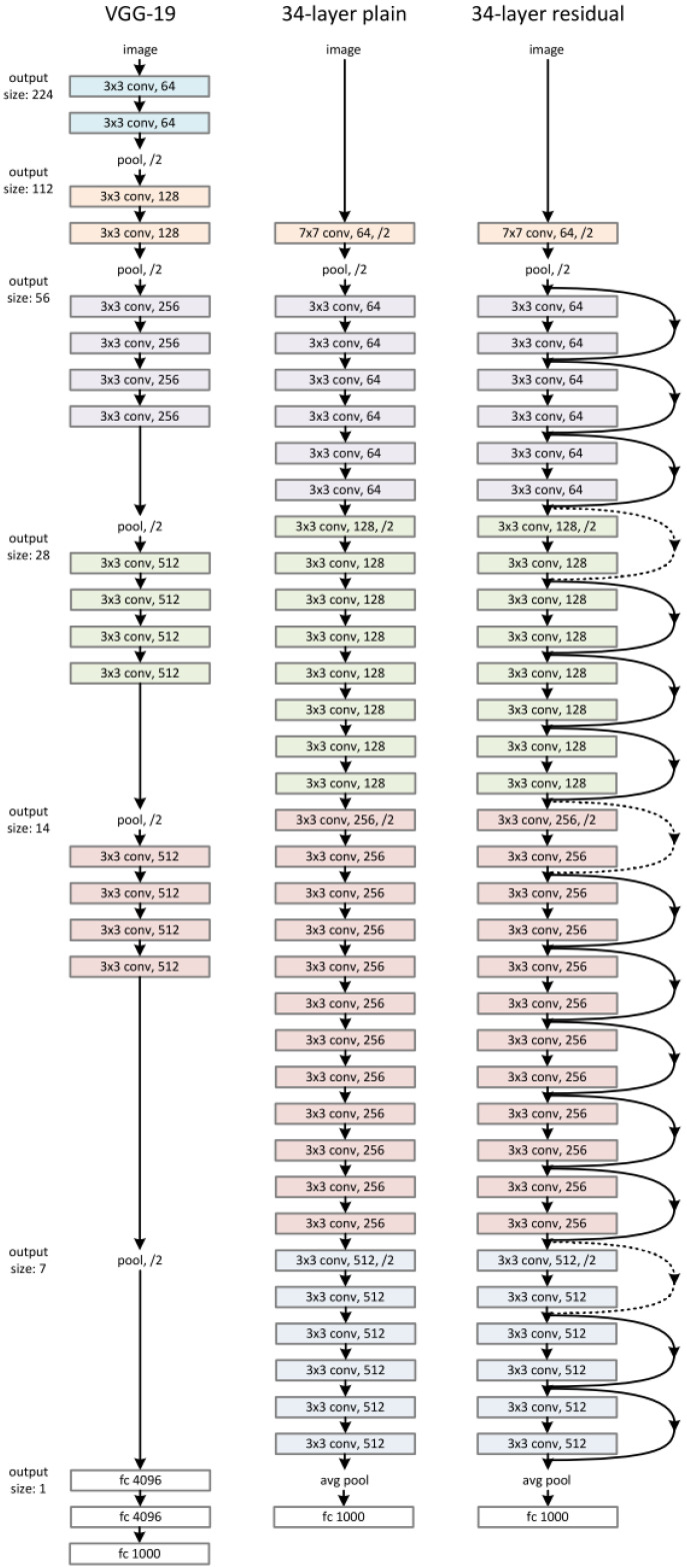
The architecture of ResNet101.

The model’s structure consists of repeated blocks of layers, each containing several convolutional layers followed by batch normalization and ReLU activation functions. Figure 1 provides a visual representation of the ResNet101 architecture, illustrating the arrangement of layers, residual blocks, and overall network structure.

In the model training protocol, we carefully selected and implemented a set of hyperparameters to optimize the performance of our deep learning model. The training process was executed over 20 epochs, allowing the model sufficient iterations to learn from the data while mitigating the risk of overfitting. We employed a batch size of 4, which balanced computational efficiency with the model’s ability to generalize from the training data. The learning rate decay was set to 1, maintaining a consistent learning rate throughout the training process. For optimization, we utilized the Adam algorithm, a popular choice in deep learning applications. The Adam optimizer was configured with a learning rate of 0.0001, providing a fine-grained approach to weight updates. The beta 1 and beta 2 parameters, which control the decay rates of the first and second moment estimates, were set to 0.9 and 0.999, respectively, adhering to commonly accepted values in the field. Additionally, we opted not to use the AMSGrad variant of Adam, setting the AMSgrad parameter to false. This comprehensive setup of hyperparameters was designed to facilitate effective and efficient training of our model for the task of knee osteoarthritis detection and grading.

#### 2.3.3. Interpretability

To enhance the interpretability of our model’s predictions, we implemented the gradient-weighted class activation mapping (Grad-CAM) technique. This method provides visual explanations for the decisions made by CNN, highlighting the regions of the input image that most significantly influence the classification outcome. This approach is particularly valuable in medical imaging contexts, where understanding the rationale behind AI-driven diagnoses is crucial [7].

#### 2.3.4. AI Platform

**Figure 2:**
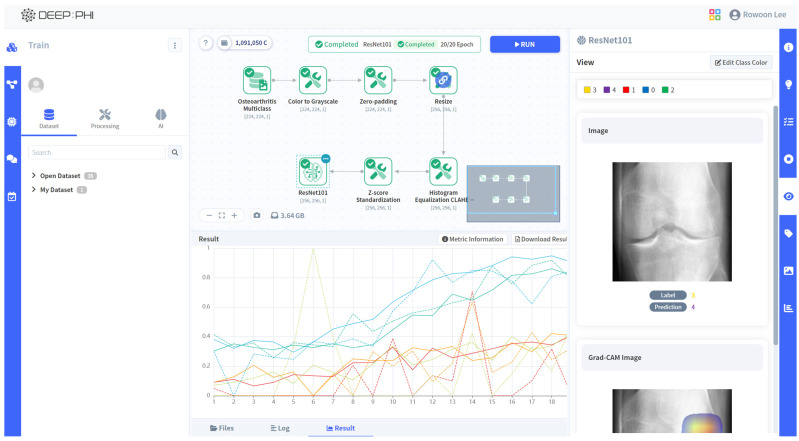
A schematic image of using the DEEP:PHI platform.

Our study leveraged a no-code AI open platform, DEEP:PHI (version 2.7.6; Deepnoid Inc., Seoul, Republic of Korea; https://www.deepphi.ai, accessed on 31 July 2024). This cloud-based solution offers a graphical user interface (GUI) that streamlines image preprocessing and AI research workflows, eliminating the need for manual coding. The platform’s drag-and-drop functionality for importing AI training images significantly expedited our research process (Figure 2).

## 3. Results

**Figure 3:**
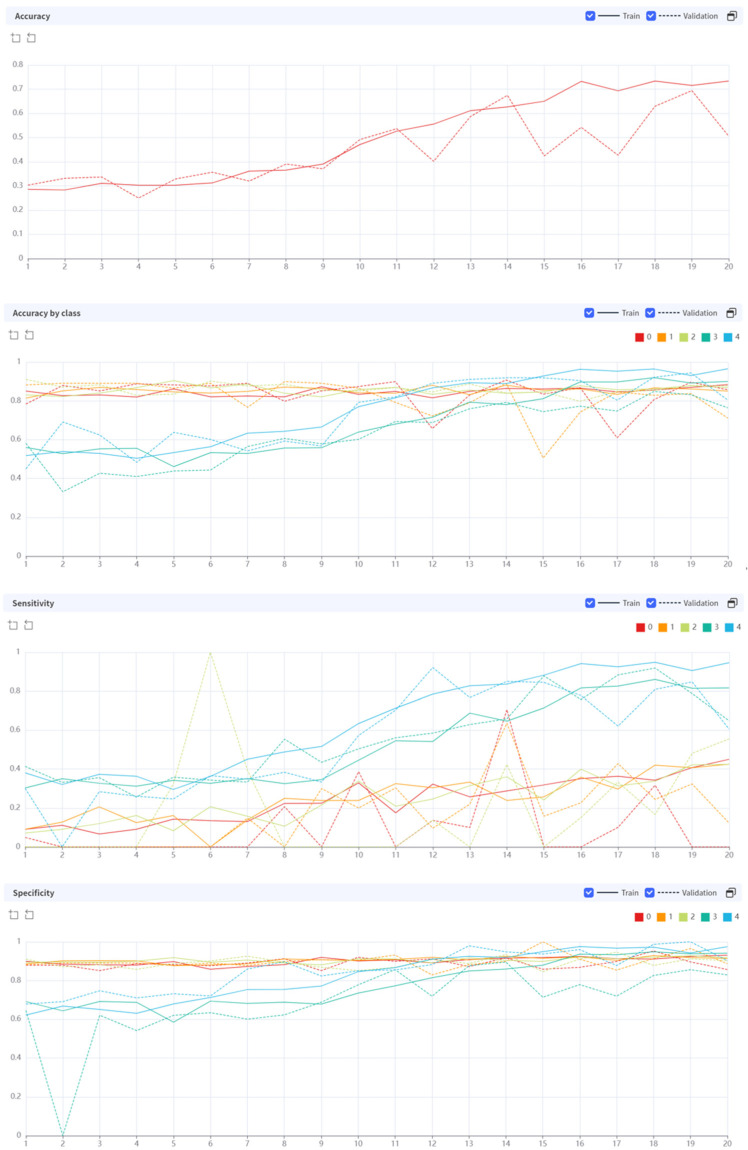
Diagnostic performance of AI algorithm during the training epoch.

While performing 20 epochs, the processing time was 42.6643 s per epoch. In the early stages of training, the AI algorithm showed a rather low diagnostic accuracy, but as the epochs were repeated, the diagnostic performance improved (Figure 3).

After 20 epochs of training, the model demonstrated varying performances across different grades of osteoarthritis. In the training set, accuracy ranged from 0.8499 to 0.9652, with the highest accuracy observed for grade 4 osteoarthritis. Sensitivity varied considerably across grades (0.4246–0.9462), with grade 4 showing the highest sensitivity. Specificity remained consistently high across all grades (0.9137–0.9748).

The validation set showed a slight overall decrease in performance compared with the training set. Accuracy in the validation set ranged from 0.7106 to 0.8764, with grade 2 osteoarthritis achieving the highest accuracy. Sensitivity varied widely (0.1219–0.8704), with grade 0 demonstrating the highest sensitivity. Specificity remained relatively high across all grades (0.8289–0.9029).

Notably, the model performed exceptionally well in identifying grade 0 osteoarthritis in the validation set, with high sensitivity (0.8704) and positive predictive value (0.9400). However, it struggled with grade 1 osteoarthritis, showing low sensitivity (0.1219) and F1 score (0.1626) in the validation set.

The model’s performance for grades 3 and 4 osteoarthritis was relatively consistent between training and validation sets, maintaining reasonable accuracy, sensitivity, and specificity.

The model’s performance for grades 3 and 4 osteoarthritis demonstrated relatively consistent and robust results between training and validation sets. For grade 3, the model achieved high accuracy in both training (0.8999) and validation (0.7640) sets. Sensitivity remained strong, with 0.8166 in the training set and 0.6484 in the validation set. Specificity was notably high in both sets (training: 0.9416, validation: 0.8289).

Grade 4 osteoarthritis showed the highest overall performance in the training set, with an accuracy of 0.9652, sensitivity of 0.9462, and specificity of 0.9748. While there was some decrease in the validation set, performance remained strong with an accuracy of 0.8033, sensitivity of 0.6050, and specificity of 0.9029.

The positive predictive value (PPV) for both grades was particularly high in the training set (grade 3: 0.8749, grade 4: 0.9502) and remained robust in the validation set (grade 3: 0.6803, grade 4: 0.7578). These results suggest that the model maintains good diagnostic capability for higher grades of osteoarthritis, even when applied to new data, though there is some expected performance decrease in the validation set. Detailed figures can be found in Table 2.

**Figure 4:**
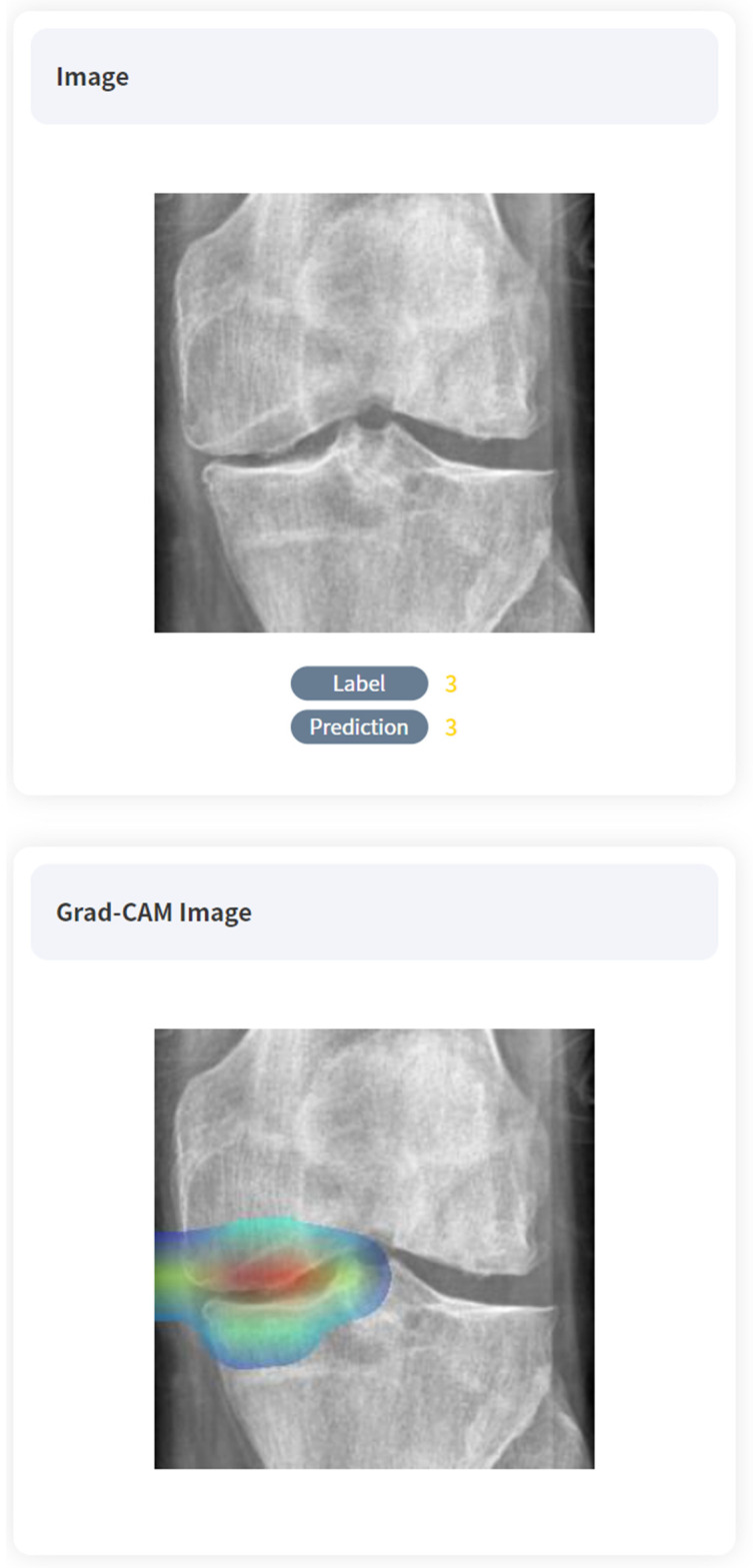
True-positive cases on AI algorithms with Grad-CAM images. The abnormal regions learned through the training can be identified by the observer through color mapping.

Referring to the Grad-CAM image, we can see that overall, the deep learning model was able to locate and grade the knee joint arthritis despite undergoing unsupervised learning. Positive samples are shown in Figure 4.

## 4. Discussion

### 4.1. Model Performance and Implications

Our model’s performance, as illustrated in the attached file, shows high accuracy in distinguishing between different grades of knee OA. This aligns with previous studies that have demonstrated the efficacy of deep learning models in medical image analysis. The use of ResNet101, a deep convolutional neural network, allowed for the extraction of complex features from knee radiographs, potentially capturing subtle indicators of OA that might be overlooked in traditional clinical assessments [8].

The high sensitivity and specificity achieved by our model are particularly noteworthy. These metrics suggest that the AI system can reliably identify both the presence and absence of OA, which is crucial for clinical decision making. The balanced performance across different OA grades indicates that the model has successfully learned to differentiate between various stages of the disease, a challenge often faced in OA diagnosis due to the subtle progression of joint space narrowing and osteophyte formation. In clinical practice, balancing sensitivity and specificity is crucial for effective OA detection. High sensitivity can lead to overdiagnosis and overtreatment, while high specificity can result in underdiagnosis and delayed interventions. Understanding these metrics helps clinicians select the best diagnostic tools and strategies, influencing treatment decisions and patient management, thereby emphasizing their importance in evaluating OA detection models.

Moreover, the model’s ability to accurately classify early-stage OA (Kellgren–Lawrence grade 1 and 2) is particularly significant. Our study highlights the potential of an AI model to enhance the early detection of knee OA. By improving early detection capabilities, such a model could enable earlier interventions, potentially altering the disease’s progression and leading to better long-term outcomes for patients. This aligns with this study’s objectives to improve patient care through innovative diagnostic tools [9].

### 4.2. DEEP:PHI No-Code AI Platform: Democratizing AI in Healthcare

Several platforms democratize access to AI by providing user-friendly interfaces, pre-built models, and drag-and-drop functionalities, making it possible for non-technical users to harness the power of AI for various applications. For example, the Clarifai platform specializes in computer vision, natural language processing, and audio recognition. It provides tools for transforming unstructured data into structured information, covering the entire AI lifecycle from data preparation to model deployment [10].

One of the unique features of our approach lies in the utilization of the DEEP:PHI no-code AI platform. This innovative platform allowed for the rapid development and deployment of our AI model without the need for extensive coding expertise. The democratization of AI tools in healthcare has been identified as a crucial step in accelerating the adoption of these technologies in clinical settings [11]. Our study provides empirical evidence supporting the viability of no-code platforms in developing sophisticated medical AI applications.

The use of DEEP:PHI addresses several critical barriers to AI adoption in healthcare. Primarily, it enhances accessibility by eliminating the need for advanced coding skills, thus making AI development accessible to a broader range of healthcare professionals and researchers. This democratization of AI development has the potential to significantly accelerate innovation in medical AI by allowing those with domain expertise but limited coding skills to contribute directly to AI development.

Furthermore, the platform’s cloud-based nature contributes to resource efficiency [12]. By reducing the need for expensive local computing infrastructure, DEEP:PHI potentially enables smaller institutions and resource-limited settings to engage in AI research and development. This could lead to a more diverse and inclusive AI development ecosystem in healthcare, potentially resulting in AI solutions that better address the needs of a wider range of healthcare contexts.

The intuitive interface of DEEP:PHI facilitates rapid prototyping, allowing for quick iteration and experimentation. This capability could potentially accelerate the pace of innovation in medical AI. The ability to quickly test and refine AI models could lead to faster development cycles and more rapid improvements in AI performance for medical applications.

Additionally, by providing a common platform for AI development, DEEP:PHI could contribute to greater standardization in medical AI research. This standardization could facilitate easier replication and validation of studies, addressing one of the key challenges in current medical AI research. The ability to more easily reproduce and build upon previous work could significantly accelerate progress in the field.

However, it is important to acknowledge that the use of no-code platforms also raises questions about the depth of understanding that developers have about the underlying AI algorithms [13]. This could potentially lead to issues with model interpretation and troubleshooting. As AI becomes more integrated into clinical decision making, it is crucial that healthcare professionals have a robust understanding of the strengths and limitations of these tools. Future research should explore how to balance the accessibility of no-code platforms with the need for the robust understanding of AI principles among healthcare professionals. This might involve developing targeted educational programs or creating more transparent and interpretable AI models.

### 4.3. Training Dynamics and Computational Efficiency

The performance of our model at epoch 20 raises interesting questions about the optimal training duration for medical AI systems. While conventional wisdom often suggests that more training leads to better performance, our results indicate that a well-designed model can achieve high accuracy relatively early in the training process. This finding has important implications for the computational resources required to develop effective medical AI tools, potentially making such systems more accessible to researchers and healthcare providers with limited computing infrastructure.

Several factors may contribute to this early convergence. The use of transfer learning, specifically the application of pretrained weights from ImageNet, may have provided a strong starting point for our model [14]. This approach allowed it to quickly adapt to the task of OA diagnosis, leveraging general image features learned from a large and diverse dataset. The quality and curation of our training data likely also played a crucial role. High-quality well-curated training data may have enabled the model to learn efficiently, reaching a good performance with fewer epochs [15].

The architecture of our chosen model, ResNet101, may be particularly well suited to the task of OA diagnosis [16]. The deep residual structure of ResNet101 allows for the efficient learning of relevant features, potentially contributing to its rapid convergence on this specific task. Additionally, the careful tuning of hyperparameters, such as learning rates, may have contributed to a rapid and stable training [17]. The optimization of these parameters is a crucial aspect of AI model development that can significantly impact training efficiency and model performance.

These findings suggest that future research in medical AI should focus not only on model accuracy but also on training efficiency. Developing strategies for rapid model convergence could significantly reduce the computational resources required for AI development in healthcare [18]. This, in turn, could potentially broaden participation in the field, allowing researchers and institutions with limited resources to contribute meaningfully to medical AI development. Such democratization of AI development in healthcare could lead to a more diverse range of AI solutions better adapted to various healthcare contexts and needs.

### 4.4. Clinical Implications and Potential Impact

The potential clinical implications of our findings are substantial and multifaceted. At its core, the accurate and efficient OA diagnosis enabled by our AI model could lead to earlier interventions, potentially slowing disease progression and improving patient outcomes. Early detection and intervention could greatly improve the quality of life for patients with OA, potentially minimizing the need for invasive treatments while enabling the administration of novel therapies with minimal adverse effects [19].

Moreover, the use of AI-assisted diagnosis could help standardize OA assessment across different healthcare settings. This standardization could reduce inter-observer variability, enhancing the consistency of the care that patients receive regardless of where they are treated [20]. Such consistency is crucial for both individual patient care and for larger-scale clinical research efforts.

In primary care settings, our AI model could serve as a valuable screening tool [21]. It could aid in identifying patients who may benefit from early intervention or referral to specialists [22], potentially catching cases of OA that might otherwise go unnoticed in their early stages. This could lead to more timely and appropriate care, potentially improving outcomes and reducing the overall burden of OA on the healthcare system.

For ongoing patient care, regular AI-assisted analysis of knee radiographs could provide objective measures of disease progression. This capability could be invaluable in treatment planning and evaluation, allowing clinicians to more accurately track the effectiveness of interventions and adjust treatment plans as necessary. The objective nature of AI-based assessments could also facilitate more informed discussions between healthcare providers and patients about disease progression and treatment options.

In the realm of clinical research, our model could be used to efficiently grade large numbers of knee radiographs in clinical trials. This could potentially accelerate research into new OA treatments by streamlining the process of patient assessment and follow-up in clinical studies. The consistency and speed of AI-based assessments could lead to more robust and faster-moving clinical trials, potentially speeding up the development of new therapies for OA.

The potential applications of our AI model also extend to telemedicine [23]. In remote or underserved areas, AI-assisted diagnosis could provide valuable support to healthcare providers who may have limited experience with OA diagnosis. This could help to address healthcare disparities by providing high-quality diagnostic support in areas where specialist care may be less accessible.

Finally, the visualizations generated by our AI model could serve as powerful educational tools for patients. By providing clear visual representations of their condition, these tools could help improve patients’ understanding of their disease, potentially leading to better treatment adherence and more engaged patient participation in their care [24]. This improved patient education and engagement could have far-reaching effects on overall treatment outcomes.

It is crucial to note, however, that while AI tools show great promise, they should be viewed as aids to clinical decision making rather than replacements for clinical judgment [25]. The integration of AI into clinical practice should be thoughtfully carried out, with ongoing evaluation of its impact on patient outcomes and healthcare processes [26]. The goal is to enhance, rather than replace, the critical thinking and expertise of healthcare professionals [27].

### 4.5. Limitations and Challenges

While our study demonstrates promising results, it is important to acknowledge its limitations and the challenges that remain in translating these findings into clinical practice. One primary concern is the generalizability of our model. Our AI was trained and tested on a specific dataset, and its performance may vary when applied to different patient populations or imaging protocols. To address this, further validation on diverse multi-center datasets is necessary to ensure the model’s robustness across various clinical settings and patient demographics.

The interpretability of our model’s decision-making process remains a challenge, despite its high accuracy. While ResNet101 demonstrates impressive performance, understanding how it arrives at its diagnoses is not straightforward. This “black box” nature of deep learning models can be a barrier to trust and adoption in clinical settings [28,29]. Due to the “black box” nature of AI predictive models, this study allows for verification of where the model has performed staging by using Grad-CAM images. For example, it becomes possible to review whether appropriate grading was conducted in an incorrect location or incorrect grading in an appropriate location. This not only impacts clinicians’ clinical judgment but is also expected to play an important role in educational processes. One study highlighted the challenge of developing AI models that can accurately answer clinical questions based on medical images [30]. Developing methods to explain the model’s predictions in clinically meaningful terms is crucial for building trust among healthcare providers and facilitating wider adoption of AI tools in clinical practice.

Integrating AI tools into existing clinical workflows presents another set of challenges. Successfully incorporating our AI model into day-to-day clinical practice requires careful consideration of factors such as user interface design, integration with electronic health records, and the impact on clinical efficiency. It is essential to design AI tools that complement and enhance existing workflows rather than disrupt them.

As AI tools become more prevalent in healthcare, navigating the evolving regulatory landscape for medical AI devices will be crucial. Clear guidelines for the validation and approval of AI-based diagnostic tools are still developing, and staying abreast of these regulations while ensuring our tool meets all necessary standards will be an ongoing challenge.

The use of AI in healthcare also raises important ethical questions that must be carefully addressed [31,32]. Issues surrounding data privacy, potential algorithmic bias, and the changing nature of the doctor–patient relationship in an AI-augmented clinical environment are all areas that require thorough consideration and ongoing dialogue among stakeholders.

The Osteoarthritis Initiative (OAI) dataset has several limitations that may affect the interpretation and generalizability of AI training based on its findings. The dataset’s inclusion criteria can introduce bias, as it primarily includes individuals with existing knee OA or risk factors, potentially limiting its applicability to the broader population. Demographic differences, such as age and BMI, compared with other populations further impact representativeness. The variability in data collection methods and the focus on radiographic evidence at baseline also constrain the dataset’s applicability to individuals without visible signs of OA.

The long-term performance stability of AI models in clinical settings remains an open question. As clinical practices and imaging technologies evolve, the performance of our model may drift over time. Developing strategies for the ongoing monitoring and updating of AI models in clinical settings will be essential to ensure their continued accuracy and relevance.

Lastly, while AI tools have the potential to improve efficiency, their implementation also involves costs. Rigorous economic evaluations are needed to determine the cost-effectiveness of AI-assisted diagnosis in various healthcare settings [33]. These evaluations should consider not only the direct costs of implementing and maintaining AI systems but also the potential long-term savings from improved early diagnosis and treatment.

### 4.6. Future Research Directions

Our study opens several promising avenues for future research, each with the potential to further advance the field of AI-assisted OA diagnosis and management. One particularly promising direction is the exploration of multimodal integration in AI models. There is a growing need for noninvasive methods in animal-based experimental medical studies [34], molecular therapeutic strategies [35], and even in surgical procedures for patients with intracranial hemorrhage [36]. Future studies could investigate the integration of several data sources, such as clinical history, physical examination findings, and other non-invasive imaging modalities like MRI, alongside radiographic images. This multimodal approach could potentially enhance the accuracy and clinical utility of AI-assisted OA diagnosis, providing a more comprehensive view of the patient’s condition.

Longitudinal studies represent another crucial area for future research. Investigating the ability of AI models to predict OA progression over time could provide invaluable insights for treatment planning and patient management. Such studies could help clinicians better understand the likely course of a patient’s condition and tailor interventions accordingly.

As data privacy concerns continue to be a critical issue in healthcare, exploring federated learning approaches could allow for the development of more robust and generalizable models while addressing these concerns. Federated learning techniques could enable the training of AI models across multiple institutions without the need to centralize sensitive patient data, potentially leading to more diverse and representative training datasets.

The development of explainable AI techniques specific to OA diagnosis is another important area for future work. Creating methods to generate clinically meaningful explanations for AI predictions is crucial for building trust and facilitating adoption in clinical settings. This could involve developing visualization techniques that highlight the specific radiographic features influencing the AI’s diagnosis, making the decision-making process more transparent to clinicians.

Investigating how AI tools can best augment, rather than replace, clinical decision making is an important area for future research. This includes studying the impact of AI on clinical workflows and decision-making processes and developing best practices for human-AI collaboration in healthcare settings.

AI tools like DEEP:PHI hold significant potential to improve over time, driven by ongoing innovation in AI technology and its growing integration within the healthcare sector. Comparative studies across different AI architectures and no-code platforms could provide valuable insights into the strengths and limitations of various approaches to medical AI development, helping guide the selection of suitable tools and techniques for diverse clinical applications. As AI-assisted diagnosis becomes more widespread, understanding patient perspectives on these technologies is crucial. Future research should focus on exploring patient attitudes toward AI in healthcare and identifying effective strategies for communicating AI’s role in healthcare decision making

Finally, investigating the potential of AI tools to address healthcare disparities, particularly in resource-limited settings, could have significant implications for global health. Research into how AI can be effectively deployed in diverse healthcare contexts, including low-resource environments, could help ensure that the benefits of these technologies are widely and equitably distributed.

These future research directions highlight the dynamic and evolving nature of AI in healthcare. As we continue to explore and refine these technologies, it will be crucial to maintain a balance between innovation and rigorous scientific evaluation, always keeping the goal of improving patient care at the forefront of our efforts.

## 5. Conclusions

This study demonstrates the effectiveness of ResNet101, implemented through the DEEP:PHI no-code AI platform, in accurately diagnosing and grading knee osteoarthritis (OA) from plain radiographs. Our AI model showed high performance in distinguishing between different OA grades, particularly in early-stage detection, which is crucial for timely intervention and improved patient outcomes.

The use of a no-code platform like DEEP:PHI represents a significant step towards democratizing AI in healthcare, enabling rapid development and deployment of sophisticated medical AI applications without extensive coding expertise. This approach addresses key barriers to AI adoption in healthcare, including accessibility and resource efficiency.

While our findings are promising, we acknowledge the need for further validation on diverse datasets and improved model interpretability. Future research should focus on multimodal integration, longitudinal studies, and investigating the impact of AI on clinical workflows.

In conclusion, this study lays the groundwork for integrating AI into rheumatology and orthopedic practice. However, it is crucial to view these AI tools as aids to clinical decision making rather than replacements for clinical judgment. As we continue to refine these technologies, maintaining a balance between innovation and rigorous scientific evaluation will be essential, always prioritizing the goal of improving patient care.

## Figures and Tables

**Table 1:** Dataset proportion and class ratio.

Proportion			
Training Set	Validation Set	Test Set	Total
717 (47.0%)	405 (26.5%)	404 (26.5%)	1526 (100%)
Class Ratio			
Index	Class Name	Count (Ratio)	
0	Grade 0	604 (39.6%)	
1	Grade 2	275 (18.0%)	
2	Grade 3	403 (26.4%)	
3	Grade 4	200 (13.1%)	
4	Grade 1	44 (2.9%)	

**Table 2:** Detailed diagnostic performance of training set and validation set (epoch 20/20).

	Class	Accuracy	Sensitivity	Specificity	PPV	NPV	F1 Score
Train	0	0.8833	0.4507	0.9306	0.4155	0.9393	0.4324
	1	0.8499	0.4255	0.9137	0.4255	0.9137	0.4255
	2	0.8680	0.4246	0.9180	0.3690	0.9339	0.3949
	3	0.8999	0.8166	0.9416	0.8749	0.9112	0.8448
	4	0.9652	0.9462	0.9748	0.9502	0.9728	0.9482
Validation	0	0.8567	0.8704	0.8567	0.9400	0.9999	0.9038
	1	0.7106	0.1219	0.8868	0.2439	0.7714	0.1626
	2	0.8764	0.5555	0.9027	0.3191	0.9611	0.4054
	3	0.7640	0.6484	0.8289	0.6803	0.8076	0.6639
	4	0.8033	0.6050	0.9029	0.7578	0.8199	0.6728

## Data Availability

Data used in the preparation of this article were obtained from the Osteoarthritis Initiative (OAI) database, which is available for public access at https://data.mendeley.com/datasets/56rmx5bjcr/1, accessed on 3 May 2024, reference number [5]. [Osteoarthritis Initiative (OAI) database] [https://data.mendeley.com/datasets/56rmx5bjcr/1] [5].
